# Suppression of neutrophil accumulation in mice by cutaneous application of geranium essential oil

**DOI:** 10.1186/1476-9255-2-1

**Published:** 2005-02-10

**Authors:** Naho Maruyama, Yuka Sekimoto, Hiroko Ishibashi, Shigeharu Inouye, Haruyuki Oshima, Hideyo Yamaguchi, Shigeru Abe

**Affiliations:** 1 Teikyo University Institute of Medical Mycology, 359 Otsuka, Hachioji, Tokyo 192-0395, Japan; 2 Department of Bioengineering, Faculty of Technology, Teikyo University, 1-1, Toyosato-dai, Utsunomiya, Tochigi 320-0003, Japan

## Abstract

**Background:**

Previous studies suggested that essential oils suppressed the adherence response of human neutrophils *in vitro *and that intraperitoneal application of geranium oil suppressed the neutrophil accumulation into peritoneal cavity *in vivo*. Usually, essential oils are applied through skin in aromatherapy in inflammatory symptoms. The purpose of this study is to assess the effects of cutaneous application of essential oils on the accumulation of neutrophils in inflammatory sites in skin of mice.

**Methods:**

Inflammation with accumulation of inflammatory cells was induced by injection of curdlan, a (1→3)-β-D-glucan in skin or peritoneal cavity of mice. Essential oils were applied cutaneously to the mice immediately and 3 hr after intradermal injection of curdlan. The skin with inflammatory lesion was cut off 6 hr after injection of curdlan, and the homogenates were used for myeloperoxidase (MPO: a marker enzyme of neutrophil granule) assay.

**Results:**

The MPO activity of the skin lesion induced by curdlan was suppressed dose-dependently by cutaneous application of geranium oil. Other oils such as lavender, eucalyptus and tea tree oils also suppressed the activity, but their activities seemed weaker than geranium. Juniper oil didn't suppress the activity

**Conclusion:**

Cutaneous application of essential oils, especially geranium oil, can suppress the inflammatory symptoms with neutrophil accumulation and edema.

## Background

Aromatherapy is a folk medicine using essential oils. Recently the clinical use of essential oils has expanded worldwide to include therapy against various kinds of inflammatory diseases, such as allergy, rheumatism and arthritis. These activities have mainly been recognized through clinical experience, especially through skin application via massage and ointment, but there have been relatively little scientific study on their biological actions.

Several investigators have suggested that tea tree [1,2] and lavender oils [3] suppressed allergic symptoms through the suppression of histamine release [4,5] and cytokine production [6]*in vitro *and *in vivo*. Moreover, in human, skin application of tea tree oils was reported to suppress the edema induced by intradermal injection of histamine [7]. However, very few reports [8,9] are available on the inhibitory effect of essential oils on the accumulation of inflammatory cells, which is a histological character recognized in chronic inflammatory diseases.

In earlier papers, we reported that the essential oils: lemongrass, geranium and spearmint suppressed the adherence response of human neutrophils *in vitro *[10], and that the intraperitoneal administration of geranium oil to mice lowered neutrophil recruitment into the peritoneal cavity induced by a chemotactic agent, casein injection *in vivo *[9]. Since essential oils are frequently applied through skin in aromatherapy for inflammatory symptoms, we believed that anti-inflammatory effects of the cutaneous application of these oils should be investigated in animal experiments to obtain practically valuable knowledge in this field. In the present study, we investigated the *in vivo *effects of cutaneously applied essential oils, especially geranium oil, to mice on inflammatory reactions including the accumulation of neutrophils in skin, which was induced by curdlan, a linear (1→3)-β-D-glucan known as a stimulating substance common in fungi.

## Methods

### Essential oils

The essential oils used are listed in Table 1 with their manufacturer and main constituents. Table 1 also indicates literature references that show clinical use related to inflammatory symptoms [11-13]. Essential oils were purchased from Hyperplants, Ltd. (Tokyo, Japan). The constitution of the geranium oil was determined by gas chromatography in this laboratory [14] using a GC apparatus (Model 353B, GL Sciences, Tokyo) equipped with a DB-5 column (0.5 mm × 30 m; J&W Scientific, Folsom, LA, USA), and was shown to contain about 24 % β-citronellol, 10 % citronellyl formate and 7 % geraniol and others.

**Table 1:** Essential oils, main constituents, their sources and manufacturer

Essential oil	Parent plant	Main constituents	Manufacturer of the oil	References for clinical use
Eucalyptus	*Eucalyptus glogulus*	1,8 – cineole	Sanoflore (France)	11
Tea tree	*Melaleuca alternifolia*	terpinen-4-ol	Sanoflore (France)	12,13
True Lavender	*Lavandula angustifolia*	linalool	Sanoflore (France)	11–13
Geranium Bourbon	*Perargonium asperum*	geraniol, β-citronellol	Sanoflore (France)	11–13
Juniper	*Juniperus communis*	α-pinene	Sanoflore (France)	11–13

For intraperitoneal injection, these essential oils were diluted to 2.5, 5 % solution by 2.5 % dimethyl sulfoxide (DMSO) in saline and 50 μl of Tween 20 was added to 4 ml of the essential oil solution. For cutaneous application, each essential oil was diluted to 5, 10, 20 and 50 % in DMSO.

### Agents

Curdlan, a (1→3)-β-glucan preparation purified from the culture fluid of *Alcaligenes faecalis*, was purchased from Wako Pure Chemical Industries, Ltd.(Osaka, Japan), and suspended in 10 mg/ml in saline for intradermal injection and in 5 mg/ml for intraperitoneal injection. Hexadecyltrimethylammonium bromide (HTAB), human myeloperoxidase (MPO), and tetramethylbenzidine (TMB) were purchased from Sigma-Aldrich Japan (Tokyo). Polyoxyethylene(20) sorbitan monolaurate (Tween 20) was from Wako Pure Chemical Industries, Ltd.. Prednisolone injection (10 mg/ml) was from Mitaka Pharmaceutical, Ltd. (Tokyo). Dulbecco's phosphate-buffered saline (PBS) was from Invitrogen Corp. (Carlsbad, CA, USA) and stored at 4°C. Diff-Quik was from International Reagents Corp. (Hyogo, Japan). Geraniol and linalool were from Wako Pure Chemical Industries, Ltd.. Terpinen-4-ol and β-citronellol were from Tokyo Kasei Kogyo Co., Ltd. (Tokyo). Hair remover, anchone^® ^was from Imju Co., Ltd. (Tokyo).

### Animals

All animal experiments were performed according to the guidelines for the care and use of animals approved by Teikyo University. Six week-old female ICR mice (Charles River Japan, Inc., Kanagawa, Japan) were used for all animal experiments except the one using 6 week-old female HR-1 hairless mice (Hoshino Laboratory Animals, Saitama, Japan). The photoperiods were adjusted to 12 hr of light and 12 hr darkness daily, and the environmental temperature was constantly maintained at 21°C. The mice were kept in cages housing 4–6 animals and were given ad libitum access to food and water.

### Leukocyte accumulation in peritoneal cavity

Fur in the dorsal region of mice (n = 5), approximately 20 × 50 mm square, was removed on day -3. The animals wore neck collars on day -2 to prevent their licking of the essential oils from the skin. On day 0, 200 μl of curdlan suspension (5 mg/ml) was injected intraperitoneally. A negative control group of mice was injected with 200 μl of saline instead of curdlan suspension. Immediately and 3 hr later, 100 μl of 20 % geranium oil in DMSO was dropped on the dorsal skin and gently spread over the fur-removed area using a glass spreader. To determine the number of leukocytes, mice were sacrificed with carbon dioxide 6 hr after curdlan injection. Three ml of PBS was then injected into their peritoneal cavity, and 2 ml of exudates were taken from the cavity to collect leukocytes. After centrifugation at 350 × g at 4°C for 5 min, the precipitate was suspended in 2 ml of PBS containing 10% heat-inactivated fetal calf serum (PBS solution). The numbers of leukocytes were measured by an electric cell counter; Celltac (Nihon Kohden Corporation, Tokyo). This leukocyte suspension was used for Diff-Quik staining and MPO assay as described below.

### Diff-Quik staining

Neutrophils recovered from the peritoneal cavity were fixed on slide glass by cytocentrifugation and stained by Diff-Quik as described previously [15]. Briefly, the leukocyte suspension was diluted to about 1 × 10^6 ^cells/ml. Two hundred μl of the suspension was poured into a plastic tube attached to a slide glass and cytocentrifuged at 75 × g for 5 min, the slide glasses were then stained by Diff-Quik. Percentage ratio and the number of neutrophils were calculated by counting the neutrophil number of more than 50 leukocytes/sample.

### Myeloperoxidase(MPO) assay for leukocyte suspensions

The MPO assay was based on the method of De Young et al. [16] and partly modified. One ml of the leukocyte suspension was centrifuged at 620 × g at 4°C for 2 min. The precipitate was suspended in 1 ml of 80 mM sodium phosphate buffer, pH5.4, containing 0.5% HTAB (0.5% HTAB solution), freeze-thawed 3 times and centrifuged at 1400 × g at 4°C for 5 min. Triplicate 30 μl samples of resulting supernatant were poured into 96 well microtiter plates. For assay, 200 μl of a mixture containing 100 μl phosphate buffered saline, 85 μl 0.22 M sodium phosphate buffer, pH5.4, and 15 μl of 0.017 % hydrogen peroxide were added to the wells. The reaction was started by the addition of 20 μl of 18.4 mM TMB•2HCl in 8 % aqueous dimethylformamide. Plates were stirred and incubated at 37°C for 3 min and then placed on ice where the reaction was stopped by addition to each well of 30 μl of 1.46 M sodium acetate, pH3.0. The MPO value was evaluated by measuring the absorbance of samples at 620 nm (OD value) and being converted it into MPO values per mouse. The MPO activity was expressed by relative values calculated by the following formula: (MPO value recovered from oil-treated mice)/(MPO value recovered from control mice) × 100 (%)

### Skin preparation from mice with intraperitoneal injection of essential oils

Fur in the abdominal region of mice (n = 5–6) was removed on day -3. On day 0, 50 μl of curdlan suspension (10 mg/ml) in saline was injected intradermaly in the abdominal skin of mice. Immediately and 3 hr after curdlan injection, 200 μl of the diluted geranium oil solution was injected intraperitoneally. A dose of 2.5 % solution corresponded to 5 μl of pure oil. The control group of mice was received 200 μl of 2.5 % DMSO solution. One hundred μl/flank × 2 of prednisolone was injected subcutaneously to another active control group of mice 1 hr before curdlan injection, instead of essential oil. All mice were sacrificed with carbon dioxide 6 hr after curdlan injection. Skin was cut off in a 6 mm diameter area, weighed and placed in 1.05 ml of 0.5 % HTAB solution, and stored at -20°C until assay. The average weight of the skin was calculated as a parameter of edema.

### Skin preparation from mice after cutaneous application of essential oils or its components

Fur in the abdominal and dorsal regions of mice (n = 15 for 5–20 μl geranium oil in experiment shown in Fig 3(b), n = 3–6 for another experiments) except hairless mice (n = 4–5) was removed on day -3. The animals wore neck collars on day -2 to prevent their licking essential oils from the skin. On day 0, 50 μl of curdlan suspension (10 mg/ml) in saline was injected intradermaly to mice. Immediately and 3 hr later, 100 μl solution of a designated concentration of the essential oil or its components was dropped on the dorsal skin and gently spread over the fur-removed area using a glass spreader. A dose of 20 % solution corresponded to 20 μl of pure oil. The control group of mice was applied 100 μl of DMSO. Their skin preparations were obtained 6 hr after curdlan injection as described above.

### Myeloperoxidase(MPO) assay for skin homogenate

Frozen samples were thawed at room temperature and homogenized for 45 sec at 0°C by Polytron (Kinematica AG, Lucerne, Switzerland). The homogenates were poured into sampling tubes and centrifuged at 12000 × g at 4°C for 15 min. The resulting supernatants were used for MPO assay as described above. The MPO value per each skin sample was calculated.

### Statistical analysis

**Figure 1:**
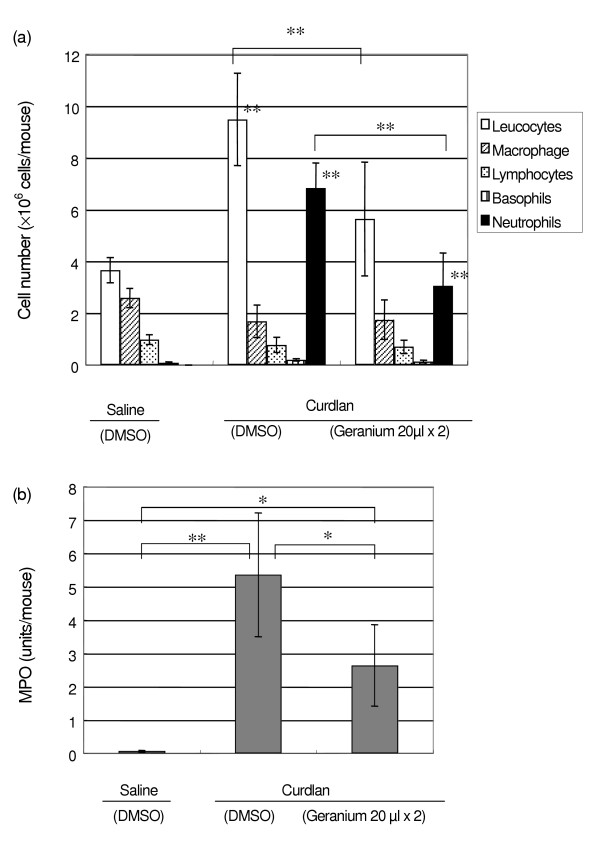
Effects of intraperitoneal injection of curdlan against neutrophil accumulation and MPO activity. Curdlan or saline was injected intraperitoneally, and immediately and 3 hr after the injection, geranium oil or DMSO was applied cutaneously. After 6 hr, leukocytes were collected for Diff-Quick staining and MPO assay. (a) The number of leukocytes and cell differentials in peritoneal exudates. (b) The MPO values in peritoneal exudates. Each value represents an average of 5 mice and the standard deviation. * p < 0.05, ** p < 0.01

**Figure 2:**
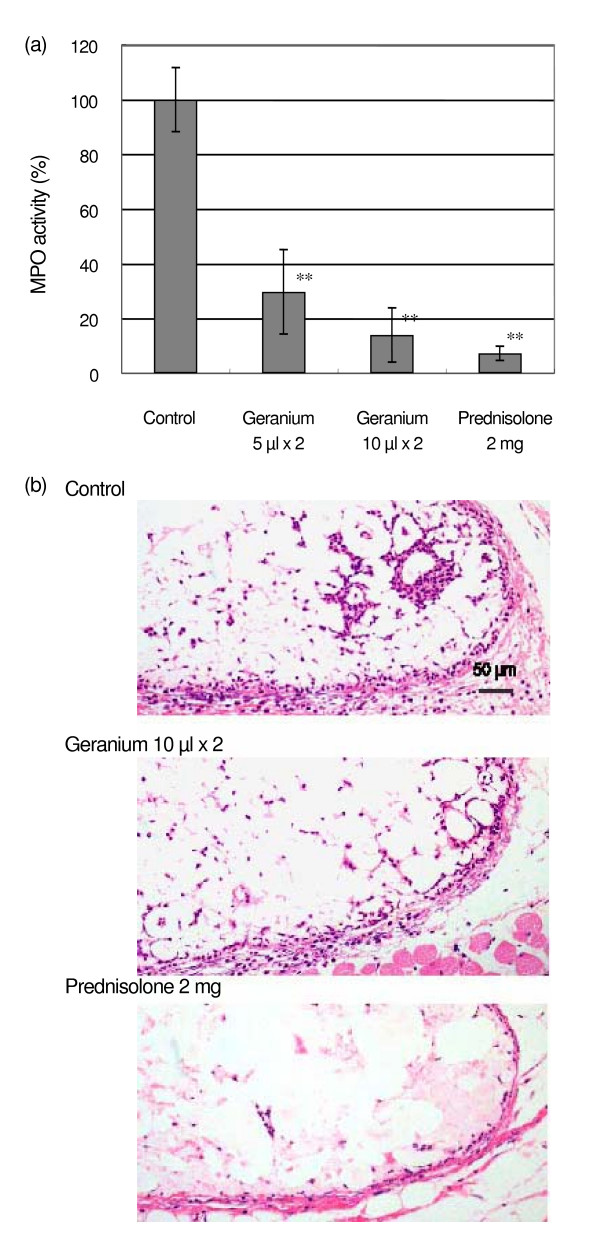
Effects of intraperitoneal injection of geranium oil on the inflammation by intradermal curdlan injection. Geranium oil was injected immediately and 3 hr after curdlan injection. Predonisolone, as positive control, was injected 1 h before curdlan injection. After 6 hr, skin was cut off for the MPO assay and histological examination. (a) The MPO activity from skin lesion. (b) Histological examination. Each value represents an average of 5–6 mice and the standard deviation. ** p < 0.01 compared with control.

**Figure 3:**
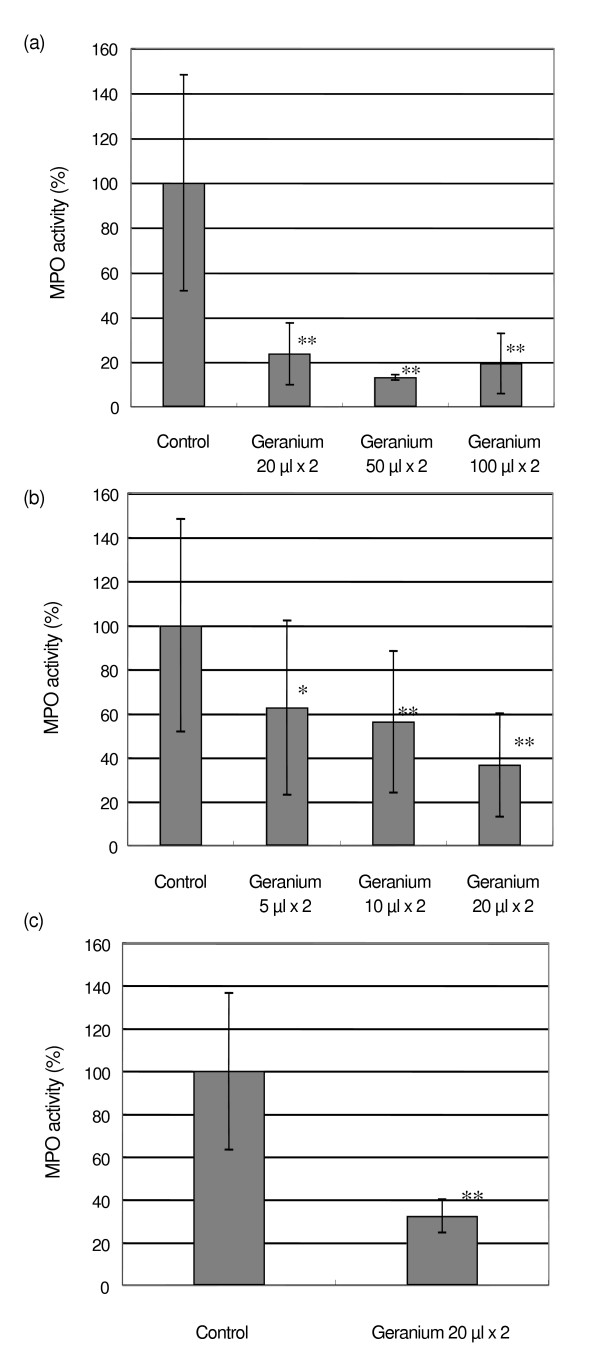
Effects of cutaneous application of geranium oil on MPO activity by intradermal curdlan injection. Geranium oil was applied immediately and 3 h after curdlan injection. After 6 h, skin was cut off for the MPO assay. (a) 20–100 μl of geranium oil was applied to fur-removed mice. (b) 5–20 μl of geranium oil was applied to fur-removed mice. Data represent the results obtained from 3 experiments. (c) 20 μl of geranium oil was applied to hairless mice. Each value represents an average of 4–5 mice for (a),(c) or 15 mice for (b), and the standard deviation. * p < 0.05, ** p < 0.01 compared with control.

**Figure 4:**
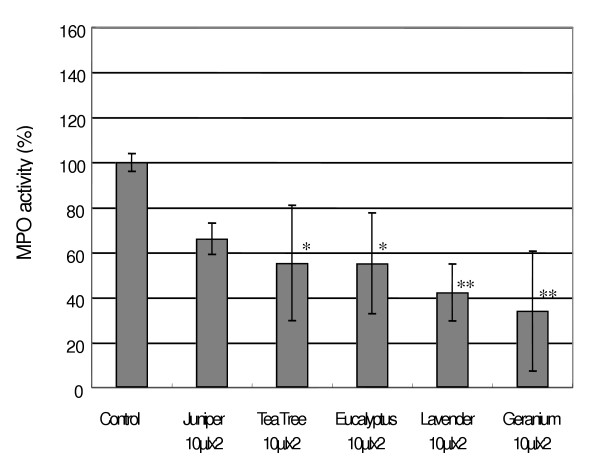
Effects of cutaneous application of essential oils on MPO activity by intradermal curdlan injection. 10 μl of essential oils was applied to fur-removed mice (n = 3–4). Each value represents an average of mice, and the standard deviation. * p < 0.05, ** p < 0.01 compared with control.

**Figure 5:**
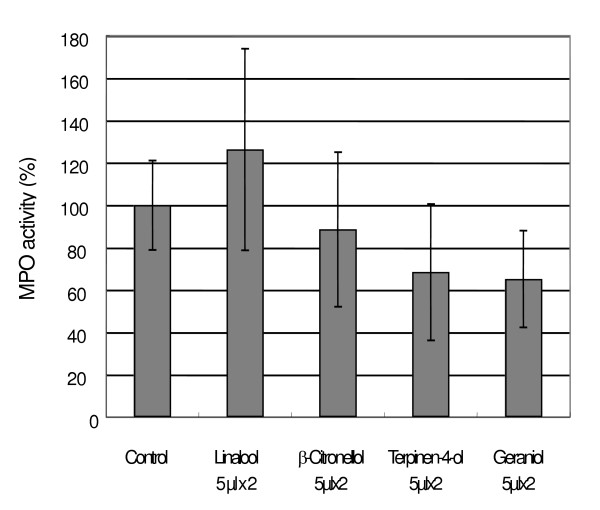
Effects of cutaneous application of essential oil components on MPO activity by intradermal curdlan injection. 5 μl of essential oil components was applied to fur-removed mice. Each value represents an average of 4–5 mice, and the standard deviation. * p < 0.05, ** p < 0.01 compared with control.

**Figure 6:**
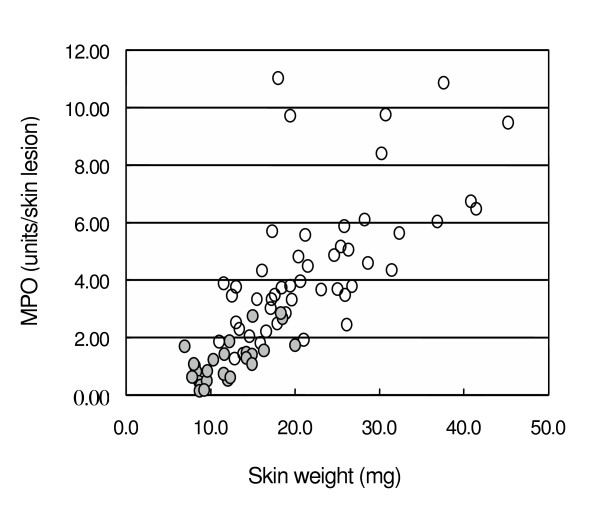
Correlation between the MPO value and skin weight. This figure is composed from data obtained from all independent experiments in which control mice and mice applied with 20 μl geranium oils were used. Open and filled circles represent control and geranium groups, respectively. r = 0.757, p < 0.0001. (n = 49 for control group, n = 26 for geranium group)

The results were expressed by the mean ± standard deviation. All statistical analysis was calculated using the StatView software. Statistical analysis was performed as follows; Students t-test for Fig 3(c), Dunnett after ANOVA for Fig 2(a), 3(a,b), 4 and 5, and Tukey-Kramer after ANOVA for Fig 1. Pearson's correlation coefficient was calculated for Fig 6.

## Results

### Inflammation of the skin by curdlan intradermal injection

Skin inflammatory response induced by intradermaly injected curdlan (0.5 mg/50 μl), was investigated first by using two parameters, the MPO value of skin homogenates and skin weight. The MPO value and skin weight of the skin lesion 6 hr after curdlan injection were 4.54 ± 2.43 units/skin lesion and 22.5 ± 8.3 mg (n = 49), which were significantly higher than those of saline injection, 0.21 ± 0.14 units/skin lesion and 8.9 ± 1.4 mg, respectively (n = 4). This indicates that curdlan injection caused neutrophil accumulation, which was monitored by increase in the MPO activity, and skin edema, which was observed by increase in skin weight.

### Correlation between myeloperoxidase(MPO) activity and neutrophil accumulation

We examined the neutrophil accumulation in the peritoneal cavity after curdlan injection microscopically and enzymatically using MPO activity. The effect of cutaneous application of geranium oil on these changes was observed.

Figure 1(a) shows the number of leukocytes and cell differentials in peritoneal exudates, which were determined using Diff-Quik staining. About 3.66 ± 0.49 × 10^6 ^leukocytes were recovered from the peritoneal cavity of saline-induced mice, and the intraperitoneal injection of 200 μl curdlan solution increased this number to 9.48 ± 1.78 × 10^6 ^cells. Neutrophils were rarely observed in peritoneal cells of saline-injected mice and the increased content of peritoneal leukocytes in curdlan-injected mice was mostly neutrophils. The MPO value of leukocyte preparation obtained 6 hr after curdlan intraperitoneal injection was 5.36 ± 1.86 units/mouse, which was significantly higher than that of the mice without curdlan (0.064 ± 0.026 units/mouse) (Fig. 1(b)).

Figure 1 also shows that compared to the curdlan control, cutaneous application of geranium oil to these mice significantly lowered the number of leukocytes and neutrophils (Fig. 1(a)) as well as the MPO value (Fig. 1(b)).

These results indicated that intraperitoneal injection of curdlan caused both the accumulation of neutrophils in the peritoneal cavity and increase of the MPO value, and that cutaneous application of geranium oil suppressed both of them. Therefore, we confirmed that the MPO activity corresponds to the number of neutrophils and that MPO activity can be used as a parameter for this number.

### Effects of geranium oil on inflammation induced by curdlan intradermal injection

The effects of geranium oil administered intraperitoneally or cutaneously on the inflammation induced by curdlan intradermal injection were examined.

At first, geranium oil was injected intraperitoneally to mice. Prednisolone was used as an active control. As shown in Fig. 2(a), administration of 2 mg per mouse of prednisolone suppressed the MPO activity to 7 ± 3 %. Similarly but to a lesser degree, 5 and 10 μl of geranium oil significantly lowered the MPO activity to 30 ± 15 and 14 ± 10 %, respectively.

From the histological examination (Fig. 2(b)), it was observed that prednisolone clearly suppressed neutrophils accumulated following curdlan injection. Geranium oil also suppressed this accumulation, however, the suppression was not as strong as by prednisolone.

In the second experiment, we examined the effect of the cutaneous administration of geranium oil. As shown in Fig. 3(a), 20, 50 and 100 μl per mouse of geranium oil application lowered the MPO activity significantly (24 ± 14, 13 ± 1 and 19 ± 13 %, respectively). In this experiment, we observed that the mice receiving 50 and 100 μl of geranium oil exhibited an unusual behavior (sedated condition with loss of normal active movement) after the second administration. Therefore, the dose of geranium oil tested was reduced to 5, 10 and 20 μl (Fig. 3(b)) and they showed significant suppression of the MPO activity (63 ± 40, 56 ± 32, and 37 ± 23 % respectively). These data depicted in Fig 3(a) and 3(b) suggest that geranium oil suppress MPO activity in a dose-dependent manner.

In the third experiment, the similar effect of geranium oil on the hairless mice was examined. In this experiment, treatment by hair remover was omitted. Figure 3(c) shows that the MPO activity in hairless mice was significantly reduced by geranium application (32 ± 8 %) as in the case of fur-removed mice. This indicated that the geranium application suppressed the fur-removed skin and normal hairless skin similarly, and the effect of the remover was negligible.

### Effects of cutaneous application of various essential oils

We compared the effects of 10 μl of various essential oils (geranium, lavender, tea tree, eucalyptus, and juniper) against the MPO activity. Although all oils except juniper oil lowered the activity significantly (Fig. 4), the inhibitory activity of geranium oil was estimated to be strongest (34 ± 27 %). On the other hand, juniper oil did not significantly suppress the activity (66 ± 7 %).

### Effects of cutaneous application of components of essential oils

We compared the activities of the main constituents of geranium, lavender and tea tree oils. As shown in Fig. 5, geraniol and terpinen-4-ol lowered the MPO activity (65 ± 23 and 68 ± 32 %, respectively), but not significantly, and linalool and β-citronellol did not lower the activity (126 ± 48 and 89 ± 37 %, respectively).

### Correlation between MPO activity and skin weight

We measured the skin weight as a parameter of edema for each experiment. The correlation between skin weight and the MPO activity was examined for all control mice and all mice applied with 20 μl of geranium oil. As shown in Fig. 6, the skin weight of each mouse closely correlated with the MPO activity (r = 0.757, p < 0.0001).

The average skin weight and the MPO value were 22.5 ± 8.3 mg and 4.54 ± 2.43 units/skin lesion for control (n = 49), and 12.0 ± 3.7 mg and 1.16 ± 0.75 units/skin lesion for the geranium group (n = 26), respectively. This indicates that geranium oil suppressed both the neutrophil accumulation and edema induced by curdlan.

## Discussion

In this study, we showed that cutaneous application of geranium oil (5–100 μl) to mice suppressed cellular inflammation induced by curdlan dose-dependently, as monitored by the MPO activity of peritoneal cavity and skin. This suppressive activity of geranium oil seemed very potent in comparison with those of other essential oils: 10 μl of lavender, tea tree, and eucalyptus oils lowered the activity significantly, but each was weaker than that of geranium oil. Juniper oil did not suppress the activity.

It was reported that MPO, a marker enzyme of neutrophil granules, can be used as a parameter of infiltration of neutrophils in various inflammatory experiments using tissues including skin [16-18]. We confirmed here that the MPO activity was closely related to the number of neutrophils which infiltrated into the peritoneal cavity after intraperitoneal injection of curdlan with or without administration of geranium oil. Histological examination of the skin, into which curdlan was injected 6 hr earlier, also showed that the degree of infiltration of inflammatory cells (perhaps neutrophils), at least qualitatively, correlated with the MPO values of the skin homogenates. These observations indicate the MPO activity can be used as a marker of neutrophil accumulation in our experiments.

As far as we know, this is the first experimental report indicating that cutaneous application of essential oils, especially geranium oil, effectively inhibited neutrophil accumulation *in vivo*. Although some irritants appeared to have anti-inflammatory activity, the action of geranium oil can not be explained by such a manner, since geranium oil did not induce neutrophil accumulation by itself as reported previously [9]. Recently, Brand et al. reported that tea tree oil inhibited histamine-induced edema [4], but did not change leukocyte infiltration in a murine contact dermatitis model [1]. In our results, cutaneous application of 10 μl of tea tree oil decreased the MPO activity in curdlan-injected skin weakly but significantly (Fig. 4), although intraperitoneal administration of the oil did not suppress the neutrophil accumulation in the peritoneal cavity [9]. Moreover, our previous report showed that geranium oil more effectively suppressed neutrophil adherence response induced by TNF-α stimulation than tea tree oil *in vitro *[10]. All these findings may suggest that geranium oil has a different type of suppressive activity for inflammation from that of tea tree oil. In order to obtain conclusive findings for quantitative differences in the anti-inflammatory activities of essential oils, we must examine their activity in a dose-dependent manner and their bioavailability based on their skin absorption.

We used curdlan, a linear (1→3)-β-glucan, as an inflammatory agent. It has already been reported that curdlan causes local inflammation and induces polymorphonuclear leukocyte accumulation [19], and that the number of neutrophils in the peritoneal cavity greatly increases 6 hr after curdlan intraperitoneal injection [15]. (1→3)-β-glucan is known to activate complements to release C5a, a neutrophil chemoattractant [20], and may induce production of chemotactic cytokines through interaction with toll-like receptors 2,6 on macrophages [21]. Therefore, we can assume that curdlan may induce neutrophil accumulation through these polysaccaride-recognition mechanisms. It is possible that geranium oil interferes with these polysaccaride-recognition steps, however, we wish to note another possibility: geranium oil may suppress neutrophil response in the accumulation step, because this oil can suppress neutrophil recruitment by casein injection *in vivo *as reported previously [9], and can strongly suppress neutrophil response by TNF-α stimulation *in vitro *[10]. Details of the mechanisms involved in the suppression of inflammation remain to be clarified.

We tested the suppressive activity for the MPO response of the main constituents (5 μl) of essential oils, geraniol and β-citronellol (geranium), linalool (lavender) and terpinen-4-ol (tea tree). Geraniol and terpinen-4-ol seemingly suppressed the activity, but the others did not. Thus, geraniol, not β-citronellol, is thought to be an active component of geranium oil. On the other hand, linalool showed no activity, although lavender oil lowered it significantly. It is possible that linalool is not an active component of lavender oil. Further examinations on the activity of various other components and their combinations are necessary to evaluate the active principles of essential oils.

The cutaneaous application of geranium oil suppressed the MPO activity dose-dependently. The GC analysis of the blood 5 min after geranium oil application showed peaks from geranium oil such as β-citronellol, which indicated some of components of the oil were absorbed in the blood circulation very quickly (data not shown). We think that the suppression by oils is done through skin absorption, although we also need to take into account the effect of inhalation of essential oil because of its high volatility. The MPO activity using hairless mice was also suppressed to about 30% by geranium oil, indicating that suppression activity was not interfered with hair remover.

We must note that in these experiments, solvent of essential oil treatments is DMSO. It is known to facilitate the permeation of some drugs. DMSO might modulate the effects of essential oils, although we reported that intraperitoneal injection of essential oils with 2.5% DMSO as solvent, which is relatively lower concentration of DMSO, lowered the neutrophil accumulation in previous study [9]. In further experiments, we need to examine the effects of essential oils using other solvents such as carrier oils.

In this study, we also examined the effect of geranium oil on the edema using skin weight as well as the MPO activity. Normal skin weight was about 8.9 ± 1.4 mg and increased to 22.5 ± 8.3 mg by curdlan injection. This difference indicates the edema by inflammation. Twenty μl of geranium oil reduced the weight to 12.0 ± 3.7 mg, indicating that the oil strongly suppresses the edema induced by curdlan injection. It is well known that tea tree and lavender oils suppress the edema induced by histamine [3,4]. As shown in Fig. 6, edema is closely correlated with the MPO activity, and geranium oil reduced both of them. The physiological meaning of this correlation should be clarified.

In aromatherapy, skin application of essential oils to limited parts of the body or in a full body massage is popular and several of these oils are used as a therapeutic treatment for inflammatory symptoms with lesional neutrophil accumulation: rheumatoid arthritis, aphthous stomatitis, and lesional bacterial or fungal infections [22]. In these cases, local application of relatively concentrated (more than 5%) oils to the lesion is effective. But full body massage with a relatively lower concentration (around 3%) of essential oils is also used for some local inflammatory conditions. These clinical usages of essential oils were established traditionally, but their pharmacological efficacies have not been fully confirmed by scientific research. Our results presented here suggest that systemic application of essential oils seems reasonable, because neutrophil accumulation and edema were suppressed through systemic application of essential oil, especially geranium oil, even though the concentration of the oil is higher than that used clinically. This suggests that some essential oils such as geranium may suppress local inflammatory symptoms through systemic skin application in human.

The therapeutic benefit of these essential oils and the roles of anti-inflammatory activity in their therapeutic actions is an urgent theme to be investigated.

## Conclusion

Cutaneous application of several essential oils, especially geranium oil, to mice suppressed the cellular inflammation induced by curdlan dose-dependently, as monitored by the MPO activity of peritoneal cavity and skin. This suggests that essential oils using in aromatherapy massage may suppresses the inflammatory symptoms related with neutrophil accumulation and edema.

## Competing interests

This work was supported in part by a grant from the Kampo Medicine Research Fund (Tokyo) and a grant (No.15590401) from the Ministry of Education. Culture, Sports, Science and Technology of Japan.

## Authors' contributions

NM participated in the design of the study, carried out the animal study and GC analysis, and wrote the manuscript. YS and HO carried out the animal study and GC analysis, and performed the statistical analysis. HI and HY helped to carry out the animal study. SI helped to carry out the GC analysis and draft the manuscript. SA conceived of the study, participated in its design and coordination, and helped to carry out the study and write the manuscript. All authors read and approved the final manuscript.
